# Alport syndrome: facts and opinions

**DOI:** 10.12688/f1000research.9636.1

**Published:** 2017-01-17

**Authors:** Clifford Kashtan

**Affiliations:** 1 Department of Pediatrics, Division of Pediatric Nephrology, University of Minnesota Medical School, Minneapolis, MN, USA

**Keywords:** Alport syndrome, collagen IV alpha-345 network, COL4A5, COL4A4, COL4A3, end-stage renal disease

## Abstract

In this commentary, I review recent advances in Alport syndrome genetics, diagnostics, and therapeutics. I also offer some opinions regarding strategies to optimize the early identification of affected individuals to promote early therapeutic intervention.

## Introduction

Alport syndrome is a rare genetic disorder of specialized basement membranes in the kidney, ear, and eye. The last five years have been eventful ones in Alport syndrome genetics, diagnostics, and therapeutics. These recent events may be of interest to those studying other rare genetic disorders, as they illustrate how more sensitive gene sequencing methods can change conventional understanding of inheritance patterns and how perceptions regarding the urgency and precision of diagnosis can change when it is demonstrated that a disease thought to be untreatable does, in fact, respond to therapy.

In this brief review, I will attempt an overview of recent events in Alport syndrome studies and their implications, at least as I see them. The reader is warned that this is not a consensus report. I have tried to indicate where a conclusion or recommendation represents personal opinion.

## Background

Alport syndrome is genetically heterogeneous, arising from mutations that impair the production, deposition, or function of the collagen IV α345 network, the major collagenous constituent of mature basement membranes in the glomerulus, cochlea, cornea, lens, and retina. The X-linked form of Alport syndrome results from mutations in *COL4A5*, which encodes the collagen IV α5 chain. Autosomal forms of Alport syndrome are caused by mutations in *COL4A3* and *COL4A4*, which are located on chromosome 2 and encode the collagen IV α3 and α4 chains, respectively. Mutations in both alleles of *COL4A3* or *COL4A4* are associated with autosomal recessive transmission, while heterozygous mutations cause autosomal dominant disease. Recently, several families transmitting mutations in two of the three genes have been described ^1^. In these families with “digenic” inheritance, transmission may not conform to Mendelian expectations.

Results of pedigree analyses and Sanger sequencing suggested relative frequencies for the X-linked, autosomal recessive, and autosomal dominant forms of approximately 80%, 15%, and 5%, respectively. However, recent studies using next-generation sequencing indicate that autosomal dominant disease accounts for a significantly greater proportion of Alport syndrome patients than was previously recognized ^2, 3^. This finding has important implications for genetic counseling and prediction of prognosis, since in autosomal dominant disease gender does not influence the risk of inheritance or the clinical phenotype. Furthermore, patients with autosomal dominant Alport syndrome generally have slower progression to end-stage renal disease (ESRD) than do patients with X-linked disease and are much less likely to have extra-renal manifestations.

Genotype-phenotype correlations in X-linked Alport syndrome were described over 15 years ago ^4– 6^. The biochemical basis for these correlations has been elucidated recently and also helps explain the difference in phenotype between X-linked and autosomal dominant disease. Monoclonal antibodies directed against the collagen IV α3, α4, and α5 chains allow expression studies using kidney and skin biopsy specimens. *COL4A5* mutations that allow expression of the collagen IV α345 network in glomerular basement membranes (GBMs), such as missense variants and small mutations that do not produce frameshifts, are associated with a slower rate of renal functional loss than are mutations that prevent expression of the network, such as major rearrangements and nonsense mutations ^7, 8^. This finding predicts that heterozygous mutations in the *COL4A3* or *COL4A4* genes, which rarely prevent GBM expression of the collagen IV α345 network, would also be associated with slower progression of Alport renal disease.

The earliest clinical manifestation of the absence of the collagen IV α345 network from basement membranes is hematuria. Deficiency of this network is associated with GBM attenuation in both animals and humans with Alport syndrome and appears to regularly allow the passage of erythrocytes through microruptures of the glomerular capillary wall, a rare event under normal conditions ^9^. The lens capsule of Alport patients, especially males, is also attenuated and lacks the mechanical integrity to maintain normal lens shape, resulting over the course of years in anterior lenticonus, or bulging of the central portion of the lens into the anterior chamber ^10– 13^. Normal expression of the collagen IV α345 network in basement membranes of the cochleae is prevented in Alport syndrome, although the mechanism through which this abnormality leads to hearing loss in Alport patients remains uncertain ^14– 16^. Findings in Alport mice suggest that the hearing loss may arise from dysfunction of the stria vascularis mediated through endothelin-1 ^14, 17, 18^, but there are no corresponding human data. On the other hand, studies of human Alport cochleae suggest that the absence of the collagen IV α345 network may disturb cochlear micromechanics ^15, 16^, but there are no supportive animal data. The pathophysiology of deafness in Alport syndrome thus remains unsettled, at least in my opinion. Inclusion of audiologic outcomes in clinical trials may provide at least empirical information about mechanisms of hearing loss in Alport syndrome.

In Alport GBM, the collagen IV α345 network is replaced by the collagen IV α112 network, which spreads from its normal subendothelial location to occupy the entire width of the GBM ^19, 20^. This change is accompanied by the ectopic appearance of laminin-211, collagen V, and collagen VI in the GBM ^19, 21, 22^. Invasion of mesangial filopodia may be responsible for deposition of these extracellular molecules in Alport GBM ^23– 25^. The Alport GBM matrix is less highly cross-linked and more susceptible to proteolytic injury than is normal GBM ^26, 27^. Altered signaling through collagen and laminin receptors results in complex cellular events, including activation of focal adhesion kinase in podocytes, endothelin-A receptor activation in mesangial cells, and glomerular inflammation ^21, 24^. Eventually, TGFβ1-mediated pathways promote glomerular and tubulointerstitial fibrosis, leading to ESRD ^28– 34^. Albuminuria appears to be an important contributor to tubular epithelial cell injury and fibrosis ^35^.

These recent findings in animal models of Alport syndrome suggest a number of potential targets for therapeutic intervention to delay renal disease progression. Attention has focused on early initiation of angiotensin blockade, which is remarkably effective in murine Alport syndrome and, according to retrospective analyses, can delay the onset of ESRD in human Alport syndrome ^36, 37^. Starting angiotensin blockade while renal function is still normal appears to be associated with the greatest impact on timing of ESRD ^36^. Whether earlier introduction of therapy, for example prior to the development of overt proteinuria, may have additional benefit is the subject of the EARLY PRO-TECT trial underway in Europe ^38^ (ClinicalTrials.gov identifier NCT01485978).

With the preceding as background, I now turn to a discussion of several issues that have recently come to the forefront of thinking about Alport syndrome and related disorders. These issues include the classification of genetic disorders of the collagen IV α345 network, evolving changes in diagnostic evaluation, and novel treatment approaches.

## Classification of genetic disorders of the collagen IV α345 network

Compared to patients with hemizygous mutations in *COL4A5* (males with X-linked Alport syndrome) and patients with mutations in both alleles of *COL4A3* or *COL4A4* (autosomal recessive Alport syndrome), patients with heterozygous mutations in these genes tend to have milder renal involvement, with ESRD occurring relatively late in life (or not at all), and are less likely to have extrarenal manifestations such as hearing loss and ocular changes. Nevertheless, heterozygous patients have a higher lifetime risk of ESRD than does the general population and appear to benefit from angiotensin blockade ^37^, raising the question of how best to ensure that these patients receive appropriate monitoring and intervention. Although this is a complex issue, it can for the sake of discussion be reduced to two questions: (1) is there a true carrier state for Alport syndrome, and (2) is thin basement membrane nephropathy (TBMN) an accurate and useful diagnostic entity?

For many years, female members of Alport families who had hematuria were considered to be carriers who were not at risk for ESRD, despite reports of ESRD in female Alport patients (discussed in 39). Thinking began to change in the early 2000s with a report by Jais and colleagues describing clinical outcomes in nearly 300 girls and women with confirmed heterozygous *COL4A5* mutations ^40^. In their cohort, the probability of developing ESRD before the age of 40 years was 12% and reached 30% by age 60. Proteinuria developed in 75% of subjects and increased the risk of developing ESRD. An additional finding was that 95% of heterozygous females had hematuria. While ascertainment bias could have inflated some of these percentages, there is no question that girls and women who have heterozygous mutations in *COL4A5* have a substantial lifetime risk of ESRD.

If one uses the conventional definition of “carrier” (“an individual that carries, but does not express, a gene for a particular recessive trait”, from *Stedman’s Medical Dictionary*, Houghton Mifflin Company, 2001), then only about 5% of women with heterozygous mutations in *COL4A5* are truly carriers and 95% are affected with X-linked Alport syndrome. The designation of a woman with a *COL4A5* mutation as a carrier creates an expectation of a benign outcome and a potential impediment to regular monitoring and early therapeutic intervention. The authors of an excellent recent review of Alport syndrome in women and girls made note of differences of opinion regarding the use of “carrier” or “affected” for X-linked Alport syndrome heterozygotes and then chose to use the term “affected” to describe these females ^41^.

A similar issue arises for people with heterozygous mutations of *COL4A3* or *COL4A4*. Approximately 40–50% of these individuals are asymptomatic with normal urinalyses and are accurately designated as carriers of autosomal recessive Alport syndrome ^42, 43^. Clinical outcomes among heterozygous individuals with hematuria are variable, ranging from normal renal function throughout life to chronic kidney disease to ESRD ^44^. As with *COL4A5* heterozygotes, proteinuria appears to be a risk factor for chronic kidney disease and ESRD ^45^. How should we classify these individuals so as to promote regular monitoring and early therapeutic intervention? My proposal is that we describe them as having autosomal Alport syndrome. This would require the education of patients, physicians, and payers and recognition that progression to ESRD in Alport syndrome is not inevitable.

This leads us to the topic of so-called TBMN. Patients with hematuria may be classified as having TBMN if thinning of the GBM is the predominant pathological finding on renal biopsy. Although many patients with hematuria and thin GBM follow a benign course, others exhibit progressive renal disease ^45, 46^. GBM thinning may be found in young males with X-linked Alport syndrome, females of any age with X-linked Alport syndrome, males and females with autosomal recessive Alport syndrome, and males and females with heterozygous mutations in *COL4A3* and *COL4A4* ^47^. GBM thinning may also be found in patients with hematuria who have no detectable pathological alterations in *COL4A3, COL4A4,* or *COL4A5* ^48^. Other genetic loci for hematuria associated with GBM thinning have yet to be identified, although a recent report described a *COL4A1* frameshift mutation in a family with autosomal dominant hematuria, GBM thinning, kidney cysts, and progressive kidney disease ^49^. Thus, TBMN is a pathological description that does not, in and of itself, allow accurate prediction of prognosis or inheritance in an individual patient. Assigning this diagnosis may lead to deficient follow-up and inaccurate genetic counseling. For these reasons, I think that TBMN has become an obsolete diagnosis. My proposal is that patients with hematuria, thin GBM, and a mutation in *COL4A3, COL4A4,* or *COL4A5* have a form of Alport syndrome and that patients without such mutations have “hematuria with thin GBM”. Patients given a diagnosis of “hematuria with thin GBM” would need regular (e.g. annual) monitoring of blood pressure and urine protein excretion to identify those who may benefit from treatment.

## Evolving changes in diagnostic evaluation

Sanger sequencing of the *COL4A3, COL4A4,* and *COL4A5* genes progressed from a research procedure with indefinite turn-around times in the 1990s to commercial testing with predictable results reporting in the 2000s. More recently, next-generation sequencing of these genes has been adopted by commercial and hospital-based laboratories, accelerating mutation identification while lowering the cost of sequencing ^2, 3^. These innovations are modifying the diagnostic evaluation of patients and families with suspected Alport syndrome.

Prior to the advent of easily available sequencing, the diagnosis of Alport syndrome relied on clinical diagnostic criteria supplemented with pedigree data and tissue studies ^50, 51^. Clinical criteria include hematuria, hearing loss, and ocular changes. Pedigree data consist of family history of hematuria, deafness, and ESRD. Tissue studies include electron microscopy of kidney biopsy specimens supplemented by immunohistochemical assessment of the expression of collagen IV α chains in the kidney (or in skin biopsy material) ^7, 52– 55^. Informed application of these tools provided reliable but not perfectly accurate diagnosis of X-linked and autosomal recessive Alport syndrome. Where sequencing is readily available, clinical and pedigree data can be used to select patients and families for genetic testing, potentially obviating the need for diagnostic tissue studies. Next-generation sequencing of *COL4A3, COL4A4,* and *COL4A5* before tissue biopsy would be reasonable when suspicion of Alport syndrome is high, e.g. when the patient is a male with hematuria who has an extensive family history of hematuria, deafness, and ESRD. However, nephrologists, especially those caring for children, are often confronted with patients who have hematuria but no extra-renal abnormalities or positive family history. In this patient group, a diagnosis such as IgA nephropathy, which can be made only by kidney biopsy, is at least as likely as Alport syndrome, if not more so. Collagen IV gene sequencing in this setting may unnecessarily delay diagnosis and appropriate intervention while adding unneeded expense. If renal biopsy is suggestive or diagnostic of Alport syndrome, gene sequencing can then be used to confirm the diagnosis, establish inheritance, and predict prognosis based on genotype. I would argue that this approach is reasonable under present circumstances of access to sensitive genetic testing in the United States. The use of next-generation sequencing or whole-exome sequencing in the initial evaluation of monosymptomatic and oligosymptomatic glomerular hematuria, prior to tissue biopsy, may become more widespread as access to these methods spreads and costs decrease, as is already occurring in the United Kingdom and Europe. Identification of sensitive and specific biomarkers for Alport syndrome could contribute to targeted application of genetic analysis ^56, 57^.

## Novel treatment approaches

The current standard of care for patients with Alport syndrome is angiotensin blockade in those with overt proteinuria ^58, 59^. Treatment at an earlier stage (microalbuminuria) should be considered in males with X-linked Alport syndrome who have a *COL4A5* genotype associated with early progression to ESRD or family history of ESRD before age 30, and in males and females with autosomal recessive Alport syndrome ^58^. Data showing that initiation of angiotensin blockade while renal function is still normal delays the onset of ESRD have not been broken down by genotype, so we do not yet know if certain genotypes are associated with better response to treatment.

It is possible that early angiotensin blockade could be sufficient to prevent ESRD in Alport males with missense mutations in *COL4A5* and in people with heterozygous mutations in *COL4A3, COL4A4,* and *COL4A5.* In many patients, angiotensin blockade is likely to provide only an incremental benefit, and additional interventions will be required to attain the goal of preventing ESRD. Studies in transgenic Alport mice have demonstrated beneficial effects of a variety of therapeutic interventions, although none have been as effective in prolonging survival as angiotensin-converting enzyme inhibition (reviewed in 60). Interventions can be generally grouped as attempts to reverse the genetic defect ^61– 64^, normalize altered glomerular cell signaling and behavior ^24, 65^, or impede TGFβ1-mediated fibrosis ^66^.

Pharmaceutical companies have become interested in Alport syndrome in the past several years, a long-awaited and very welcome development. A phase II study of anti-microRNA-21 treatment of Alport syndrome will begin enrolling patients 18 years of age or older with glomerular filtration rates (GFRs) of 45–90 ml/min/1.73 m ^2^, with a primary outcome of change in the rate of decline in GFR (ClinicalTrials.gov Identifier NCT02855268). In transgenic Alport mice, anti-microRNA-21 treatment reduced glomerular inflammation and impaired renal fibrotic pathways ^29^. A phase II/III study of bardoxolone ^67^ therapy for Alport syndrome patients with chronic kidney disease has been announced but was not listed on ClinicalTrials.gov at the time this review was submitted.

Among the most attractive features of angiotensin blockade therapy for Alport syndrome are the safety and accessibility of these agents ^68^. They are inexpensive, widely available, and effective through oral administration, so barriers to treatment should in theory be minimal. While I am an enthusiastic supporter of the development of novel treatments for Alport syndrome (just see my Competing Interests), I am concerned that pricing issues may limit the availability of the next generation of Alport syndrome treatments. Pharmaceutical companies must recoup development costs, investors deserve returns on their investments, and financial profit is expected. At the same time, the primary goal of innovative therapy should be to achieve the maximal benefit for patients, and realizing this goal may be impeded if access is limited by costs.
